# Epithelial-Mesenchymal Transition Is a Critical Step in Tumorgenesis of Pancreatic Neuroendocrine Tumors 

**DOI:** 10.3390/cancers4010281

**Published:** 2012-03-08

**Authors:** Volker Fendrich, Katja Maschuw, Jens Waldmann, Malte Buchholz, Johannes Rehm, Thomas M. Gress, Detlef K. Bartsch, Alexander König

**Affiliations:** 1 Department of Surgery, Philipps University Marburg, Baldingerstraße, Marburg D-35043, Germany; 2 Department of Gastroenterology and Endocrinology, Philipps-University Marburg, Baldingerstraße, Marburg D-35043, Germany

**Keywords:** EMT, E-cadherin, snail, slug, twist, Rip1Tag2, PEG

## Abstract

The transcription factors Snail, Slug and Twist repress E-cadherin and induce epithelial-mesenchymal transition (EMT), a process exploited by invasive cancer cells. In this study, we evaluated the role of EMT in the tumorgenesis of neuroendocrine tumors of the pancreas (PNETs) *in vitro*, *in vivo* and human tumor specimen. Expression of EMT markers was analyzed using immunohistochemistry and real-time PCR. For *in vitro* studies, BON-1 cells were analyzed regarding expression of EMT markers before and after transfection with siRNA against Slug or Snail, and cell aggregation assays were performed. To asses *in vivo* effects, Rip1Tag2 mice were treated with vehicle or the snail-inhibitor polythlylenglykol from week 5-10 of age. The resected pancreata were evaluated by weight, tumor cell proliferation and apoptosis. Snail and Twist was expressed in 61 % and 64% of PNETs. This was associated with loss of E-cadherin. RT-PCR revealed conservation of the EMT markers Slug and Snail in BON-1 cells. Transfection with siRNA against Slug was associated with upregulation of E-cadherin, enhanced cell-cell adhesion and inhibition of cell proliferation. Snail-inhibition *in vivo* by PEG was associated with increased apoptosis, decreased tumor cell proliferation and dramatic reduced tumor volume in Rip1Tag2 mice. The presented data show that EMT plays a key role in tumorgenesis of PNETs. The activation of Snail in a considerable subset of human PNETs and the successful effect of Snail inhibition by PEG in islet cell tumors of transgenic mice provides first evidence of Snail as a drug target in PNETs.

## 1. Introduction

Pancreatic neuroendocrine tumors (PNETs) are fascinating tumors with an annual incidence of 1 per 100,000 people [1]. PNETs present as either functional tumors, causing specific hormonal syndromes like Zollinger-Ellison-Syndrome (ZES) or organic hyperinsulinism, or as non-functional pancreatic tumors (NF-PNETs). The natural history of PNETs is highly variable. Small, benign neoplasms such as 90% of all insulinomas are readily curable by surgical resection. Most other functional and all NF-PNETs have a less favorable prognosis. Survival is determined by local invasiveness and liver metastases [2]. The increased motility and invasiveness of cancer cells in the first phase of metastasis are reminiscent of epithelial-mesenchymal transition (EMT) during embryonic development. In EMT, epithelial cells acquire fibroblast-like properties and show reduced intercellular adhesion and increased motility [3]. Numerous observations support the idea that EMT has a central role in tumour progression. During progression to metastatic competence, carcinoma cells acquire mesenchymal gene-expression patterns and properties. This results in changed adhesive properties and the activation of proteolysis and motility, which allows the tumour cells to metastasize and establish secondary tumours at distant sites [4]. During EMT, the E-cadherin promoter is frequently repressed by specific transcriptional repressors, including Snail, Slug, and Twist. Of the transcriptional repressors, the best-studied is Snail, a highly unstable protein. It is rapidly phosphorylated by glycogen synthase kinase-3β (GSK-3β) and subsequently degraded by the ubiquitin–proteasome pathway. Conversely, inhibition of GSK-3β function results in upregulation of Snail by an NF-κB-dependent pathway, loss of E-cadherin expression, and EMT. Additional protein modification further stabilizes Snail protein and promotes EMT and tumour invasion [5]. Expression of Snail in epithelial tumors increases their aggressiveness, as seen in experimentally induced breast tumors, where high Snail expression correlates with an increased risk of tumor relapse and poor survival rates in human breast cancer [6]. Recently, we were the first to show that EMT plays an important role in tumorgenesis of neuroendocrine tumors of the ileum [7] and in other endocrine tumors [8,9,10].

In the present study we now evaluated the function of EMT in PNETs by *in vitro* and *in vivo* experiments and analysis of human specimens. The results suggest that EMT plays a key role in tumorgenesis of neuroendocrine pancreatic tumors.

## 2. Material and Methods

### 2.1. Subjects

A series of 94 PNETs, including 30 insulinomas, 21 gastrinomas and 43 non-functioning endocrine pancreatic carcinomas were obtained from the tissue bank of the Department of Pathology, Philipps-University of Marburg, Germany. In patients with PNETs, Zollinger-Ellison-Syndrom (ZES) was established by clinical symptoms, an elevated fasting serum gastrin level (>125 pg/mL), a positive secretin stimulation test defined as an increase of serum gastrin concentration to >200 pg/mL together with low pH in the stomach, and a positive immunohistochemistry for gastrin in the tumor cells. The diagnosis of insulinoma required a symptomatic hypoglycaemia (<40 mg/dL) with concomitant endogenous hyperinsulinism (>20 µU/mL) during a supervised fasting test and a positive immunohistochemistry for insulin in the tumor cells. Lesions were considered as NF-PET, if there were no clinical symptoms of hormonal excess present and plasma hormone levels except those of pancreatic polypeptide were within normal limits. Malignancy for PNETs was determined on the basis of strict criteria of infiltrating growth, lymph node or distant metastases. All experimental protocols were approved by the appropriate institutional review committee and meet the guidelines of their responsible governmental agency.

### 2.2. Mice

The generation of RIP1-Tag2 mice as a model of pancreatic islet cell carcinogenesis has been previously reported [11]. The mice used in this study were males and females of the RIP1-Tag2 transgenic mouse lineage inbred in the C57Bl/6J background. All experiments were approved by the local Committees for Animal Care and Use. Animals were maintained in a climate-controlled room kept at 22 °C, exposed to a 12:12-h light-dark cycle, fed standard laboratory chow, and given water ad libidum.

### 2.3. Genotyping

For genotyping, genomic DNA was extracted from tail cuttings using the REDExtract-N-Amp^TM^ Tissue PCR kit (Sigma-Aldrich, Saint Louis, MO, USA). A PCR reaction was carried out for each animal, to test for the presence of Tag2. Primer sequences used were: TAG1 – 5’-GGA CAA ACC ACA ACT AGA ATG CAG –3’ and TAG2 – 5’-CAG AGC AGA ATT GTG GAG TGG –3’

### 2.4. Drug Treatment

Overall, 20 animals were treated from 5 to 10 weeks of age and were randomly assigned to receive either A) mock treatment consisting of intraperitoneal saline injections or B) 5% polyehylenglykol-8000 (PEG) as intraperitoneal injection. In cases, where littermates were available for drug treatment, only the first mouse was randomly assigned to one of the four given treatment groups; the second littermate was then assigned to the ‘matched’ control arm, and so forth. This scheme was chosen in order to obtain the highest possible degree of consistency and to avoid randomization bias as far as possible. PEG was dissolved in 0.9% NaCl and distributed i.p. with a dosage of 10 mg/kg body weight/day.

### 2.5. Necropsy and Assessment of Islet Cell Tumor Growth

After completion of drug treatment mice were euthanized and the pancreas was removed, weighted and inspected for grossly visible tumors and either preserved in 10% formalin solution (Sigma-Aldrich) for histology or processed for RNA extraction (see below). For evaluating a change in islet cell tumor growth, four sections from each mouse were analyzed. These sections were taken 1µm, 10 µm, 20 µm, and 40 µm from the surface of the pancreas and H&E stained. All the islets were marked and their surface measured.

### 2.6. Immunostaining

For immunolabeling, formalin-fixed and paraffin embedded archived tumor samples and corresponding normal tissues were stained as previously described (7). Concentrations and sources of primary antibodies are listed in Table 1. Briefly, slides were heated to 60 °C for 1 h, deparaffinized using xylene, and hydrated by a graded series of ethanol washes. Antigen retrieval was accomplished by microwave heating in 10 mM sodium citrate buffer of pH 6.0 for 10 min. For immunohistochemistry, endogenous peroxidase activity was quenched by 10 min incubation in 3% H_2_O_2_. Nonspecific binding was blocked with 10% serum. Sections were then probed with primary antibodies overnight at 4 °C. For immunohistochemistry, bound antibodies were detected using the avidin-biotin-complex (ABC) peroxidase method (ABC Elite Kit, Vector Labs, Burlingame, CA, USA). Final staining was developed with the Sigma FAST DAB peroxidase substrate kit (Sigma, Deisenhofen, Germany). The immunohistochemistry results for were scored as described previously [8,9,12]. 

**Table 1:** Concentrations and source of primary antibodies.

Antibody	Species	Working dilution	Source
α-Snail	Goat	1:100	Santa Cruz, Santa Cruz, CA
α -Vimentin	Goat	1.100	Santa Cruz, Santa Cruz, CA
α -E-cadherin	Rat	1:200	Zymed, S.F., CA
α -Twist	Goat	1:250	Santa Cruz, Santa Cruz, CA

### 2.7. RNA Extraction and Real-Time RT-PCR

Islets were isolated from 10-week-old RIP1-Tag2 mice by collagenase digestion of the excised pancreas, and selected based on their red, hemorrhagic appearance [13]. Visible tumors were microdissected from the excised pancreas and the surrounding exocrine tissue carefully removed. RNA extraction and cDNA synthesis were performed as previously described [14]. A portion of fresh tumor tissue was homogenized and lysed with 600 µl buffer RLT and whole RNA was extracted using the RNeasy kit (Qiagen, Hilden, Germany) with on-column DNA digestion following the standard protocol provided by the manufacturer. The mRNA was reverse transcribed into cDNA with oligo-dT primers using the Superscript 1^st^ Strand System for RT-PCR (Invitrogen, Carlsbad, CA, USA) at 42 ºC for 50 min. All PCRs were carried out on a 7500 Real Time PCR System (Applied Biosystems, Foster City, CA, USA) over 40 cycles, with denaturation for 15 sec at 95 ºC and combined annealing/extension at 60 ºC for 1 min. Following an activation step at 95 °C for 10 min, determination of mRNA expression was performed over 40 cycles with 15 seconds of denaturation at 95 °C and annealing/extension/data acquisition at 60 °C for 60 seconds using the Power SYBR^®^ Green PCR kit (Applied Biosystems). Primer sequences are available on request. Relative fold mRNA expression levels were determined using the 2(-ΔΔCt) method [15]. All reactions were done in triplicates and results are presented as means and standard errors. 

### 2.8. Cell Culture

BON-1 cells were maintained in Ham F-12 / DMEM medium (50:50, Invitrogen, Carlsbad, CA, USA) supplemented with 10% FCS, 1 mM sodium pyruvate and 2 mM L-glutamine (Sigma-Aldrich, St Louis, MO, USA). Cells were transfected with siRNA against Slug or Snail (Ambion Applied Biosystems, Austin, TX, USA) using lipofectamin 2000 (Invitrogen) at 50% confluence according to the manufacturer’s instructions. Cells were subjected to protein analysis 48 hours after transfection.

### 2.9. Protein Analysis

For protein analyses cells were lysed using radioimmunoprecipitation assay buffer (1% Triton X-100, 0.5% sodium deoxycholate, 0.1% sodium dodececyl sulfate, 150 mmol/L NaCl, 50 mmol/L Tris/HCl (pH 7.2), 10 mmol/L EDTA, 10 mmol/L EGTA) containing 400 mmol/L aprotinin, 50 mmol/L leupeptin, and 0.5 mmol/L Pefabloc (all from Roche Diagnostics, Indianapolis, IN, USA) to inhibit proteases. Twenty microgram of total lysates were subjected to SDS-PAGE analysis, blotted onto nitrocellulose and incubated with antibodies against E-cadherin (BD Bioscience, San Jose, CA, USA), Slug (Santa Cruz Biotechnology, Santa Cruz, CA, USA), Snail (Cell Signaling Technology, Danvers, MA, USA) or b-actin (Sigma-Aldrich) over night and a HRP conjugated secondary antibody. Immunoreactive proteins were visualized by enhanced chemiluminescence detection system.

### 2.10. Cell Adhesion Assay

To determine the cell-cell adhesion capacity, cell aggregation assays were performed as described previously [16]. Cell adhesion was analyzed 36h after transfection with indicated siRNA constructs. The aggregation index was determined by the ratio of aggregate numbers after 30 min of rotation and the particle number at the beginning (Ai 1/4 (N0–N30)/N0). E-cadherin dependence was proven by incubation with an anti-E-cadherin antibody (Sigma-Aldrich) which is targeting its extracellular domain. Five independent assays were performed in duplicate.

### 2.11. Luciferase Reporter Assay

At 70% confluence, Bon-1 cells were cotransfected with constructs containing fragments of the E-cadherin promoter, kindly provided by W. Birchmeier (Max-Delbrueck-Center Berlin, Germany), siRNA against Slug or control siRNA and a pRLTK vector (Promega, Mannheim, Germany) containing the renilla firefly gene. Cells were harvested 48 h after transfection and processed using the Dual Luciferase Kit (Promega) as suggested by the manufacturer’s instructions. Luciferase activity was normalized to renilla firefly activity. For statistical analysis, Student’s t-test was performed and p < 0.05 was considered significant.

### 2.12. Statistical Analysis

Log-rank test was applied to identify significant differences. Differences in the mean of two samples were analyzed by an unpaired t-test. Comparisons of more than two groups were made by a one-way ANOVA with post hoc Holm-Sidak analysis for pair wise comparisons and comparisons versus control and by Kruskal-Wallis one-way analysis of variance. P-values <0.05 were considered statistically significant. Data were analyzed using SPSS software (Version 16; SPSS, Inc., Chicago, IL, USA).

## 3. Results

### 3.1. Expression Pattern of EMT Markers in Human PNETs

**Figure 1:**
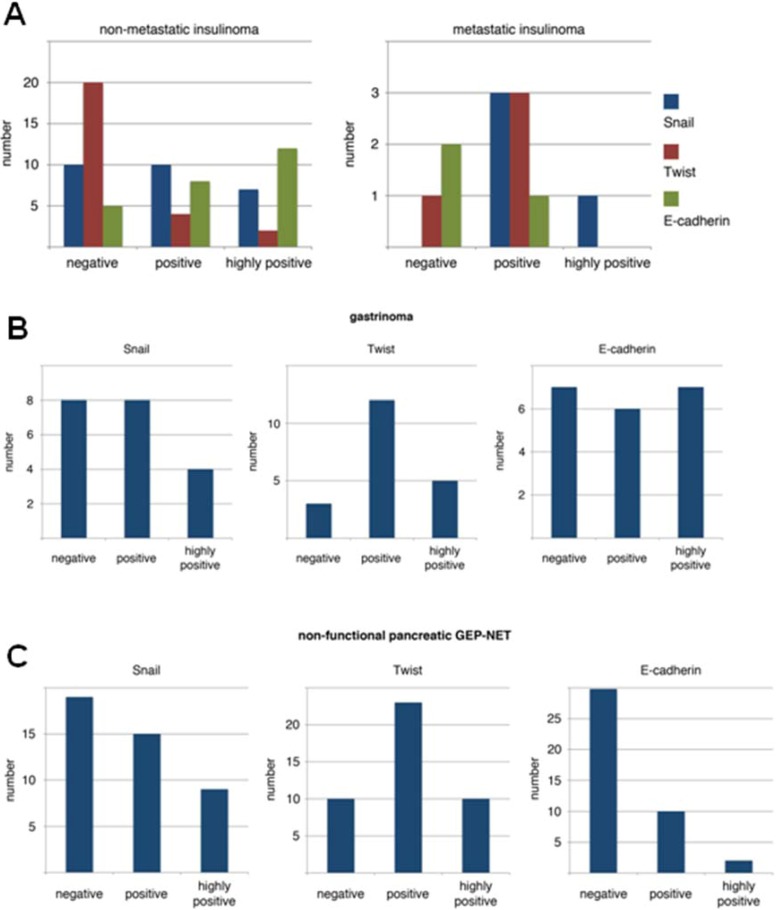
Results of immunohistochemistry for E-cadherin, Snail and Twist in (A) insulinomas, (B) gastrinomas, and in (C) non-functional PNETs.

**Figure 2:**
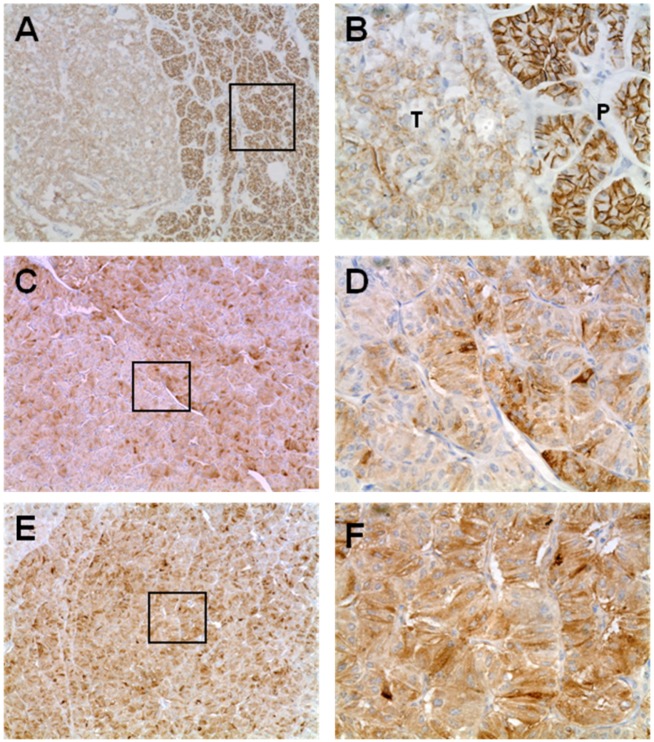
IHC staining for E-cadherin, Snail and Twist in human PNETs. Loss of E-cadherin was seen in many PNETs, as shown in (A) and (B). Note the loss of in tumor tissue (T) compared to normal membranous staining of exocrine tissue in normal pancreas (P). This loss of E-cadherin was accompanied with strong expression of Snail (C and D) and Twist (E and F), (A, C, E, 10×; B, D, F 40× magnification).

First, we evaluated the expression pattern of the EMT markers E-cadherin, Snail and Twist in human PNETs. Overall, we found loss of E-cadherin in 47/94 (50%) of all samples analyzed. When we analyzed the different entities, we found loss of E-cadherin in only 7/26 (27%) benign insulinomas, but in three of four metastatic insulinomas, in 29/43 (67%) NF-PETs, and in 8/21 (38%) gastrinomas (see Figure 1 A–C and Figure 2 A,B). Next we stained for the E-cadherin opponents Snail and Twist and found expression of Snail in 16/26 (61%) benign insulinomas, in all four metastatic insulinomas, in 24/43 (55%) NF-PETs, and in 13/21 (62%) gastrinomas.

Twist was expressed in 6/26 (23%) benign insulinomas, in three of four metastatic insulinomas, in 33/43 (77%) NF-PETs, and in 17/21 (81%) gastrinomas. Snail and Twist expression was combined in 36/94 (38%) PNETs, associated with a loss of E-cadherin (Figure 2C-F). Thirty of these 36 (80%) PNETs were classified as malignant tumors, mainly non-functional islet cell carcinomas. Next, we analyzed these expression patterns with our clinical data. Interestingly, we neither found a statistical significant correlation between loss of E-cadherin or expression of Snail and Twist and survival of the patients, nor a correlation with the occurrence of metastases in malignant PNETs.

### 3.2. Conservation of EMT Markers in BON-1 Cells

**Figure 3:**
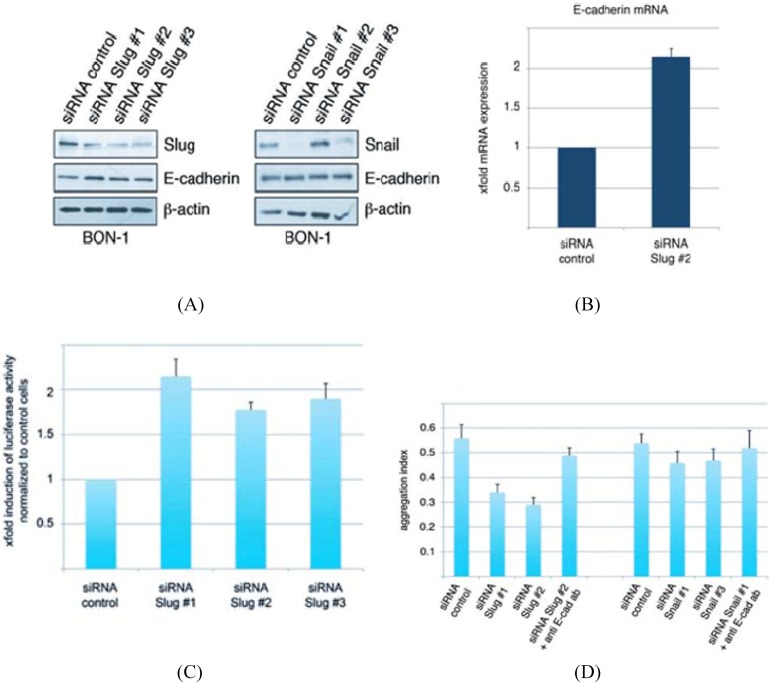
(A) Western blot analysis revealed expression of the EMT markers Snail and Slug and E-cadherin;(B) This upregulation of E-cadherin was also detectable in quantitative real-time RT-PCR analysis; (C)This effect was caused by an increased activity of the E-cadherin promoter; (D) Furthermore, we evaluated the influence of E-cadherin upregulation on the cell-cell adhesion capacity, measured by cell aggregation assay.

The pancreatic carcinoid cell line BON-1 was used for *in vitro* studies. Western blot analysis revealed expression of the EMT markers Snail and Slug and E-cadherin (Figure 3A). After knock-down of Slug and Snail by the corresponding siRNAs, we found up-regulation of E-cadherin in the BON-1 cells treated with siRNA against Slug. Interestingly, expression of E-cadherin was not altered after knock-down of Snail (Figure 3A). This upregulation of E-cadherin was also detectable in quantitative real-time RT-PCR analysis (Figure 3B). E-cadherin mRNA expression was two fold up-regulated in BON-1 cells after knockdown of Slug compared to control cells. This effect was caused by an increased activity of the E-cadherin promoter (Figure 3C). Furthermore, we evaluated the influence of E-cadherin upregulation on the cell-cell adhesion capacity, measured by cell aggregation assay (Figure 3D). We found less but larger aggregates after knockdown of Slug, but not after knockdown of Snail. This effect was reversible by the addition of an extracellular antibody against E-cadherin, demonstrating the central role of E-cadherin in this assay (Figure 3D).

### 3.3. Development of Islet Cell Tumors in Rip1Tag2 Mice

As previously described in the initial reports [11], we observed development of islet cell hyperplasia, angiogenetic islets and islet cell tumors in the Rip1Tag2 mice cohort used for our study compared to wildtype control mice.

### 3.4. Expression of EMT Markers in Rip1Tag2 Transgenic Mice

To evaluate the expression of the EMT markers in Rip1Tag2 islet cell tumors, the pancreata from these mice were harvested at week 8, and stained them for E-cadherin, Snail, Twist and the mesenchymal marker Vimentin. 

**Figure 4:**
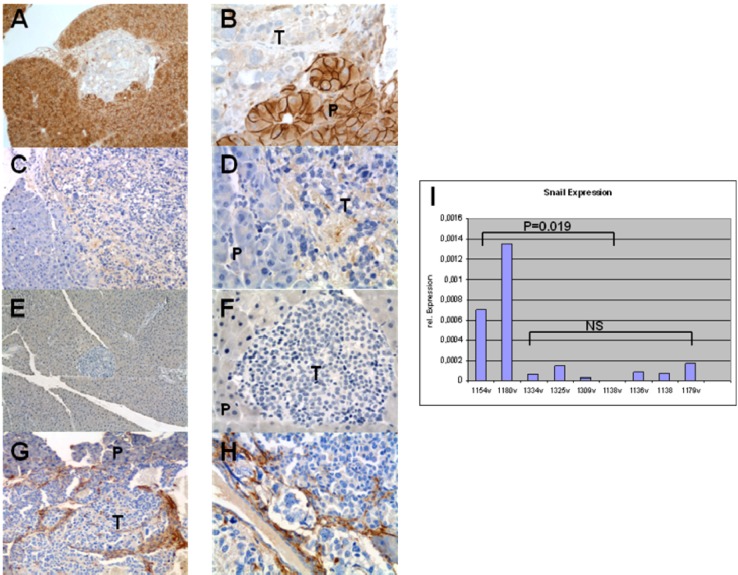
(A, B) IHC staining for E-Cadherin in tissue samples from Rip1Tag2 islet cell tumors shows loss of E-cadherin in most islet cell tumors. (C, D) IHC staining for Snail in tissue samples from Rip1Tag2 islet cell tumors, revealing slight expression of Snail in tumor tissue. (E, H) IHC staining for Twist in tissue samples from Rip1Tag2 islet cell tumors. Virtually all islet cell tumors were absent for Twist expression.(G, H) IHC staining for Vimentin in tissue samples from Rip1Tag2 islet cell tumors.Stromal cells within the tumor showed strong positive staining for the mesenchymal marker vimentin (P = normal pancreas, T = islet cell tumor). (A, C, E, G 10x; B, D, F, H 40x magnification). (T) Tumor, (P) normal pancreas. (I) Snail was down regulated in islet cell tumors by PEG. Quantitative real-time PCR on RNA demonstrates profound down-regulation of Snail in treated mice (1334v, 1325v, 1309v, 1138v, 1136v, 1138) vs. control mice (1154v, 1180v, 1179v).

As illustrated in Figure 4, islet tumor cells showed loss of expression for E-cadherin (Figure 4A and B) as seen in human PNETs (Figure 2A and B). We also found positive staining for Snail in tumor cells comparing to normal pancreatic tissue (Figure 4C and D). Twist was not found in this stages of islet cell tumor development (Figure 4E and F), whereas Vimentin (Figure 4G and H) was strongly expressed in the tumor stroma.These observations demonstrate that Snail is expressed in islet cell tumors of Rip1Tag2 mice, therefore providing a valid target for the Snail inhibitor PEG.

### 3.5. The Snail Inhibitor PEG Decreases Tumor Growth in Early Stages of Rip1Tag2 Mice

Our next step was to treat Rip1tag2 mice with the Snail inhibitor PEG [17]. In the present study, 5-week-old transgenic mice harboring only hyperplastic nonangiogenic islets were treated for 5 weeks, to the point at which tumors first began to appear in sham-treated controls. PEG dramatically reduced the tumor volume by 97% (47717 *vs.* 1432 µm^2^, p = 0.0001) in the PEG treated group. PEG had no statistically significant effect, regarding pancreas weight. The mean weight of resected pancreata in the PEG group was 0.145g *vs*. 0.149 g in the control mice (p = 0.39).

### 3.6. Snail is Down-Regulated in Islet Cell Tumors by PEG

Given the clinical and histopathological evidence that PEG decreased the tumor growth, we next explored Snail as a target of PEG-8000 in the tumor tissue of mice from the treatment group. Quantitative real-time PCR for Snail demonstrated a significant down-regulation in the islet cell tumors of Rip1Tag2 mice treated with PEG-8000 (p = 0.019; Figure 4I). This experiment confirmed our ability to achieve effective pharmacologic levels of PEG *in vivo*.

## 4. Discussion

Molecular data on PNETs has been accumulated in recent years, but the genetic basis of endocrine tumour development and progression is still poorly understood. Cancer cells undergoing EMT have lost specific target recognition and are usually equipped with autocrine loops of growth signals, mechanisms to evade apoptosis, and the potential to elicit angiogenesis for independent nutrient supply [18]. A central event in EMT is down regulation of E-cadherin, which leads to the loss of cell-cell contact and the consecutive progression of the cells towards a malignant phenotype. The transcription factor Snail is one major suppressor of E-cadherin and a strong inducer of EMT. Snail down regulates E-cadherin in different types of tumors, e.g., hepatocellular carcinomas [19], carcinomas from the esophagus, cardia, stomach [20], and colorectal carcinomas [21].

This is now the first study to show that EMT might play a key role in PNETs. Recently; we evaluated the expression pattern of EMT marker E-cadherin, Snail and Twist in different endocrine tumors [7,8,9,10] and found loss of E-cadherin and overexpression of Snail and Twist in the majority of these tumors.

In line with these results, we found expression of Snail and Twist in the majority of PNETs with highest expression in malignant PNETs (Figure 1). As expected the upregulation of these mesenchymal markers were accompanied by the loss of E-cadherin. Some years ago, a large single-centre analysis was published by Rosenau *et al*. [22] reporting on 19 patients who received liver transplantation for metastatic PNETs. They performed expression studies of Ki67 and E-cadherin and found that survival in patients with a low Ki67 and regular E-cadherin staining was significantly better than in the 12 patients with a high Ki67 or aberrant E-cadherin expression (7-year survival 100 *vs.* 0%; p = 0.007). In our study population, the expression pattern of loss of E-cadherin and/or expression of Snail and Twist was neither correlated with a shorter survival of the patients nor with the occurrence of metastases. This was somewhat surprising, but can be explained by the cumulative 10-years survival rate of 72% even in patients with malignant NF- PNETs. All patients with benign insulinomas and most of the patients with gastrinomas were alive, making it very difficult to observe a significant benefit in survival based on this expression pattern. This is also true for the development of metastases. Because our patients have such a long survival [23], virtually all patients with malignant PNETs developed metastases, so a comparison in between tumor entities was not possible.

One striking finding of our study is that inhibition of Snail by use of PEG prevented the formation of islet cell tumors in the RIP1-Tag2 transgenic mouse model. The RIP1-Tag2 transgenic mouse model serves as a general prototype of the pathways, parameters, and molecular mechanisms of multistage tumorigenesis [24]. RIP1-Tag2 mice express the oncogene SV40 T antigen under the control of the rat insulin gene promoter and display distinct stages of tumor progression: onset of hyperproliferation, induction of angiogenesis and formation of solid tumors [11,24]. Therefore, this model provides a unique opportunity to study the effect of new drug therapies in a preclinical setting [14,25]. PEG is a clinically widely used agent with profound chemopreventive properties in experimental colon carcinogenesis. Recently, Wali and colleagues showed that the epidermal growth factor receptor (EGFR) is the upstream membrane signaling molecule through which PEG initiates antiproliferative activity against Snail [17]. Furthermore, Bergmann *et al*. examined the expression of EGFR in human NPTs and found that the expression of EGFR correlated significantly with the grade of malignancy, increasing from low rates of expression in benign tumors and tumors of uncertain behavior to high rates of expression in well- and poorly differentiated endocrine carcinomas [26]. In the presented study, tumor volume could be reduced by 97% in mice treated with PEG. This result provides first evidence of PEG as chemopreventive or chemotherapeutic drug in patients NPTs. Especially, the aspect of chemoprevention in patients with Multiple Endocrine Neoplasia Type 1 (MEN1) cannot be overestimated. 

These *in vivo* findings are in line with the *in vitro* expression of EMT markes Snail and Slug in BON-1 cells (Figure 3). After knock-down of Slug and Snail by the corresponding siRNAs, we found up-regulation of E-cadherin in the BON-1 cells. E-cadherin mRNA expression was twice in BON1-cells after knockdown of Slug compared to control cells. This effect was caused by an increased activity of the E-cadherin promoter. 

## Conclusions

The presented data show that EMT might play a key role in tumorgenesis of PNETs. The activation of Snail in a considerable subset of human PNETs and the successful effect of Snail inhibition by PEG in islet cell tumors of transgenic mice provides first evidence of Snail as a drug target in PNET.
